# Supplementary material for: Long-read sequencing reveals extensive FMR1 somatic mosaicism in Fragile-X associated tremor/ataxia syndrome in human brain
Source: bioRxiv. 2025 Oct 9:2025.10.07.678589. Preprint. [Version 1] doi: 10.1101/2025.10.07.678589 (PMC12632538; doi:10.1101/2025.10.07.678589)
Supplement: Supplement 1 [file media-1.pdf]

Supplemental Table 1: Donor demographics and characterization

Identifier	Diagnosis	Age	Sex	Race	PMI	RIN	TP-PCR
FXPM5006	FXTAS	85	M	White	5	7.1	CBL, CC, BA10, BA17, BA4, BA42, PVWM, NA, Pons
FXPM5746	FXTAS	80	M	White	22	Unknown	CBL, BA22
FXPM4555	FXTAS	80	M	White	12	Unknown	CBL, BA10, NA, CC, PVWM
FXPM1011-05-RD	FXTAS	82	M	Unknown	Unknown	Unknown	CBL
FXPM1006-14-JG	FXTAS	81	M	Unknown	11	Unknown	CBL
FXPM1002-04-ER	FXTAS	87	M	Unknown	Unknown	Unknown	CBL
FXPM334-03-KC	FXTAS	81	M	Unknown	Unknown	Unknown	CBL
FXPM1007-04-LL	FXTAS	82	M	Unknown	Unknown	Unknown	CBL
FXPM1018-08-EB	FXTAS	79	M	Unknown	7.25	Unknown	CBL
FXPM1061-08-AM	FXTAS	82	M	Unknown	29	Unknown	CBL
FXPM1004-10-MG	FXTAS	77	M	Unknown	5.25	Unknown	CBL
FXPM1009-18-JF	FXTAS	72	M	Unknown	18	Unknown	CBL
FXPM1006-06-CM	FXTAS	80	F	Unknown	5	Unknown	CBL
FXPM1004-019-EA	FXTAS	89+	F	Unknown	4	Unknown	CBL
FXPM1041-08-IM	FXTAS	79	F	Unknown	Unknown	Unknown	CBL, CC
FXPM1015-09-DK	FXTAS	89+	F	Unknown	Unknown	Unknown	CBL
FXPM1008-20-RF	FXTAS	80	F	Unknown	21	Unknown	CBL, PVWM, BA10
FXS5319	FXS	71	M	White	17	6.6	CBL, BA10, PVWM
FXS1031-09-LZ	FXS	64	M	Unknown	11.5	Unknown	CBL

Supplemental Table 2: Long-read sequencing of affected individuals

	Identifier	Diagnosis	PureTarget
	FXPM5006	FXTAS	CBL, PVWM, BA10
	FXPM1008-20-RF	FXTAS	CBL, PVWM, BA10
**Intra-individual sequencing**	FXS5319	FXS	CBL, BA10, PVWM
	**Identifier**	**Diagnosis**	**PureTarget**
	FXPM5746	FXTAS	BA22
	FXPM1041-08-IM	FXTAS	CC
	FXPM1015-09-DK	FXTAS	CBL
	FXPM1033-09-MM	FXTAS	CBL
**Inter-individual sequencing**	FXS1031-09-LZ	FXS	CBL

Supplemental Table 3: qPCR primers

Primer #	Primer Name	Sequence
1	hACTBexon4-5 F	GGACTTCGAGCAAGAGATGG
2	hACTBexon4-5 R	AGCACTGTGTTGGCGTACAG
3	hGAPDHexon3-4 F	TCACCAGGGCTGCTTTTAAC
4	hGAPDHexon3-4 R	TGACGGTGCCATGGAATTTG
5	hFMR1exon8-9 F	CCTGGGGTCACTGCTATTGA
6	hFMR1exon8-9 R	TCTTCAGCAAATTCGAGAAAGCT

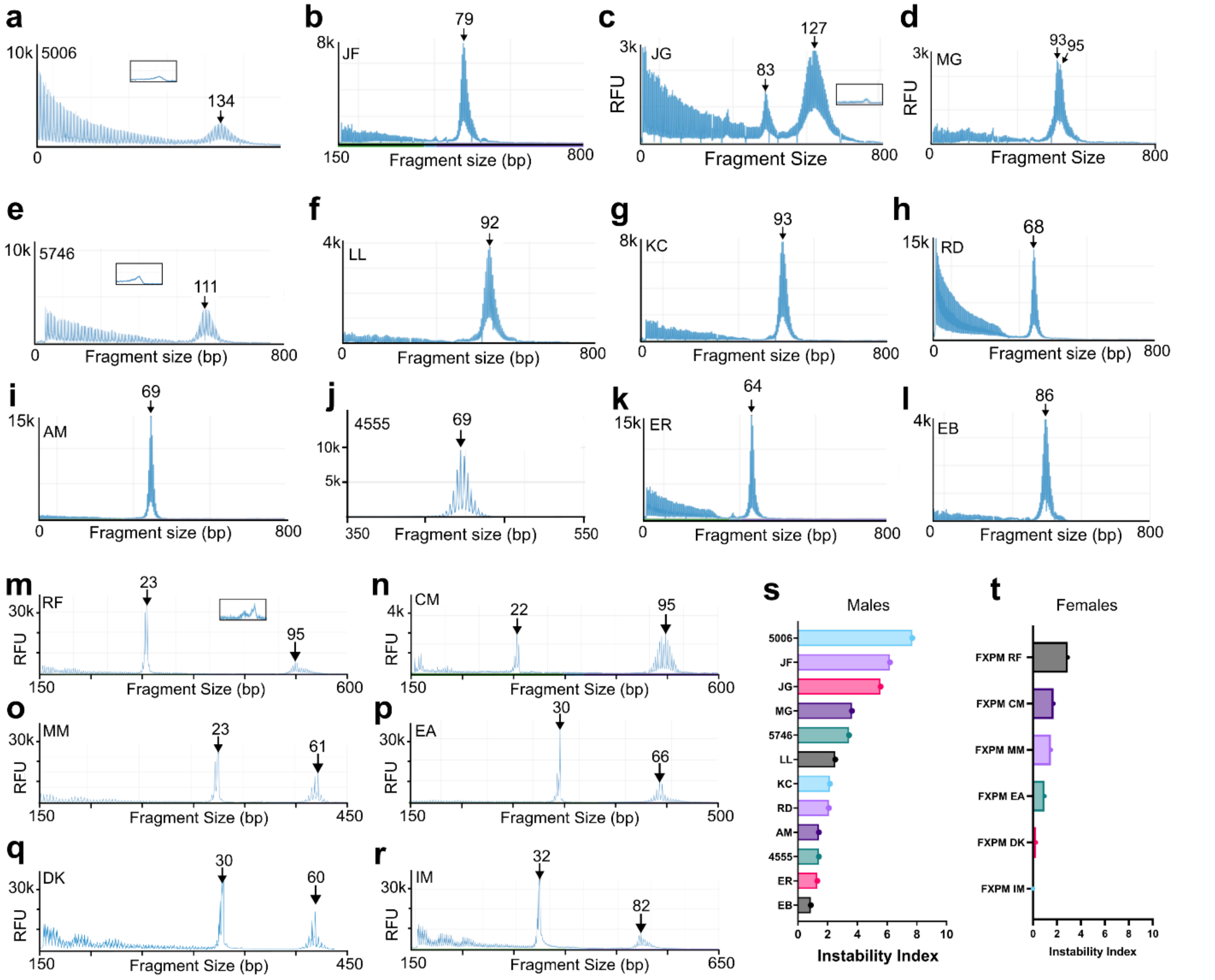

Supplemental Figure 1. *FMR1* patterns of instability in larger a FXTAS cohort. a-l) Cerebellar TP-PCR/CE of male FXTAS cohort in order of instability index value: *5006* (a), 1009-18-*JF* (b), 1006-14-*JG* (c), 1004-10-*MG* (d), *5746* (e), 1007-04-*LL* (f), 334-03-*KC* (g), 1011-15-*RD* (h), 1081-08-*AM* (i), *4555* (j), 1002-04-*ER* (k), 1018-08-*EB* (l). **m-r)** Cerebellar TP-PCR/CE of female FXTAS cohort in order of instability index value: 1008-20-*RF* (m), 1006-06-*CM* (n), 1030-09-*MM* (o), 1004-019-*EA* (p), 1015-09-*DK* (q), 1041-08-*IM* (r). s) Instability indices of male FXTAS cohort. t) Instability indices of female FXTAS cohort. Insets show zoomed in full mutation expansions > 200 bp.

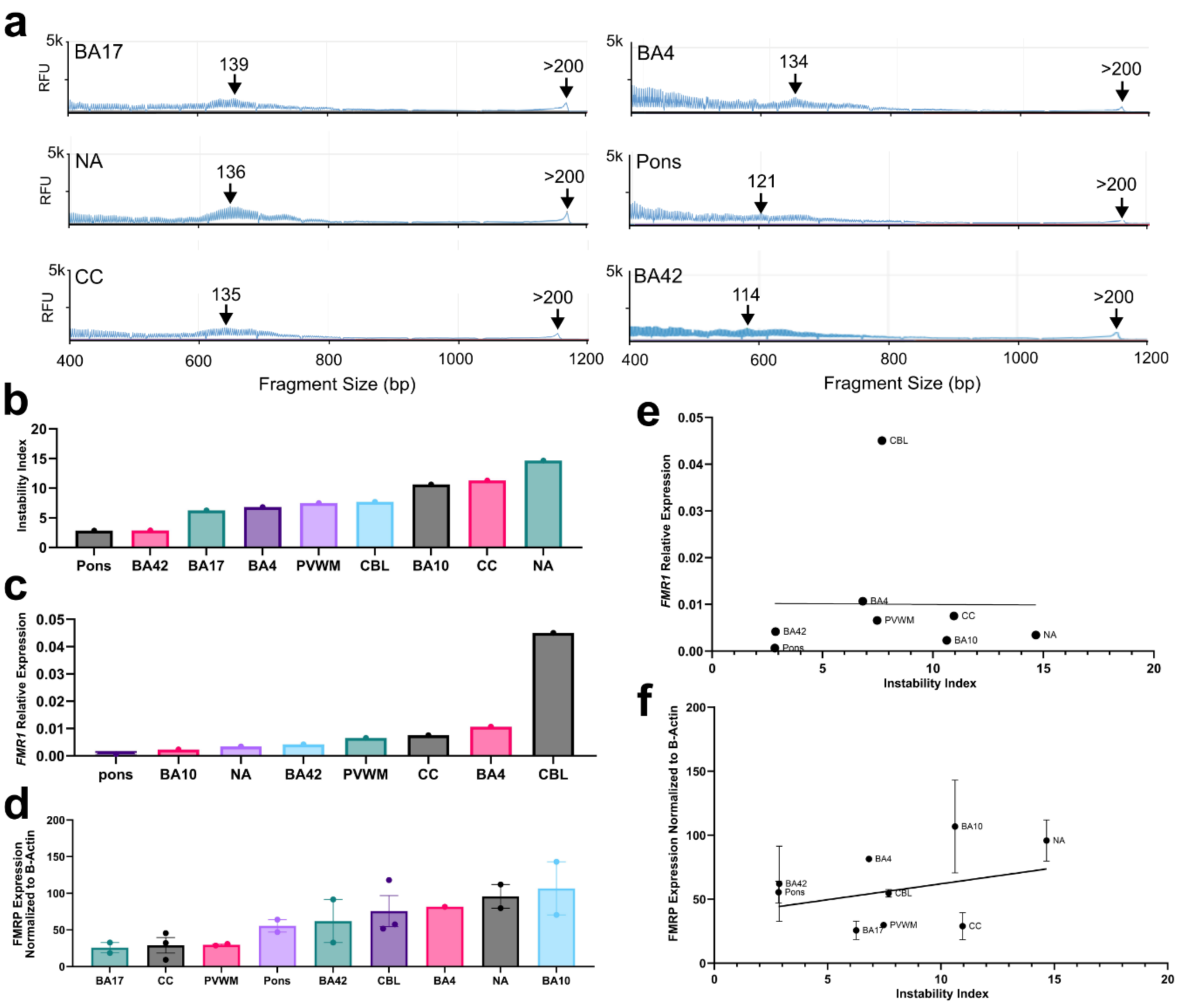

Supplemental Figure 2. *FMR1* TP-PCR in high (ii) individual. Molecular characterization from case FXPM 5006. Regions assessed: pons, BA42, BA17, BA4, periventricular white matter, cerebellum, BA10, corpus callosum, and nucleus accumbens. a) Electropherograms from brain region specific TP-PCR/CE depicting premutation allele at 139 repeats in BA17, 136 in nucleus accumbens, 135 in corpus callosum, 134 in BA4, 121 in pons, and 114 in BA42. Electropherograms for cerebellum, BA10, and periventricular white matter excluded. b) Instability index calculations. c) Bar plot depicting relative expression of *FMR1* determined via qPCR (relative expression = *ACTB* and *GAPDH*). Excludes BA17 due to poor RNA quality. d) Bar plot depicting FMRP expression normalized to Beta Actin determined by Western blot. e) Linear regression of *FMR1* relative expression against instability index across all brain regions ($\beta = -2.6x10^{-5}$, $R^2 = 5.1x10^{-5}$, $p = 0.99$). f) Linear regression of FMRP expression against instability index across all brain regions ($\beta = 2.5$, $R^2 = 0.07$, $p = 0.27$).

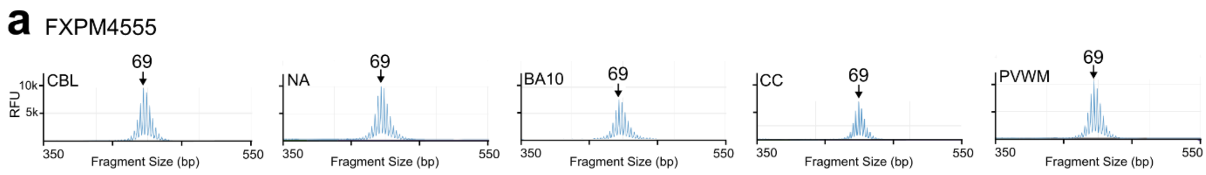

Supplemental Figure 3. Intra-individual *FMR1* TP-PCR in low (ii) individual. All molecular characterization is determined from case FXPM 4555. Region assessed: cerebellum (CBL), nucleus accumebens (NA), BA10, corpus callosum (CC), and periventricular white matter (PVWM). Premutation allele detected at 69 repeats in all regions.

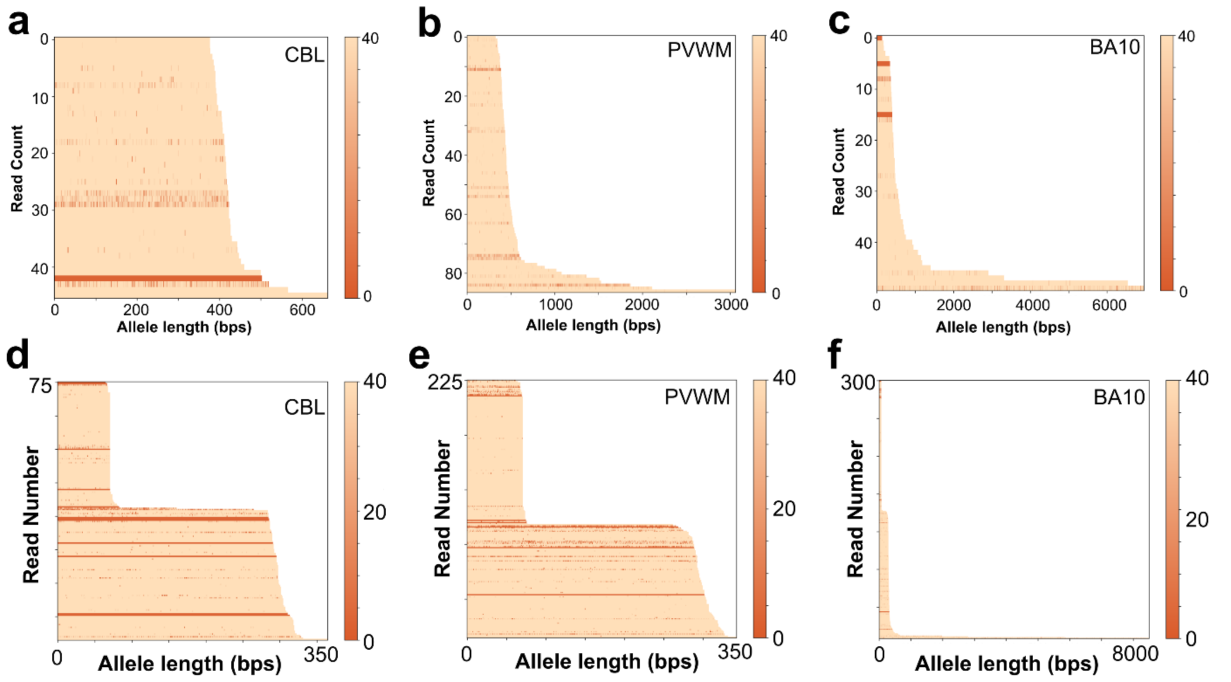

Supplemental Figure 4. Intra-individual patterns of *FMR1* methylation in FXTAS. a-c) Base-call quality plots for cerebellum (a), periventricular white matter (b), BA10 (c) for male FXPM 5006. Base call quality scale is 0 = poor, 40 = high quality. **d-f)** Base-call quality plots for cerebellum (a), periventricular white matter (b), BA10 (c) for male FXPM 1008-20-RF.

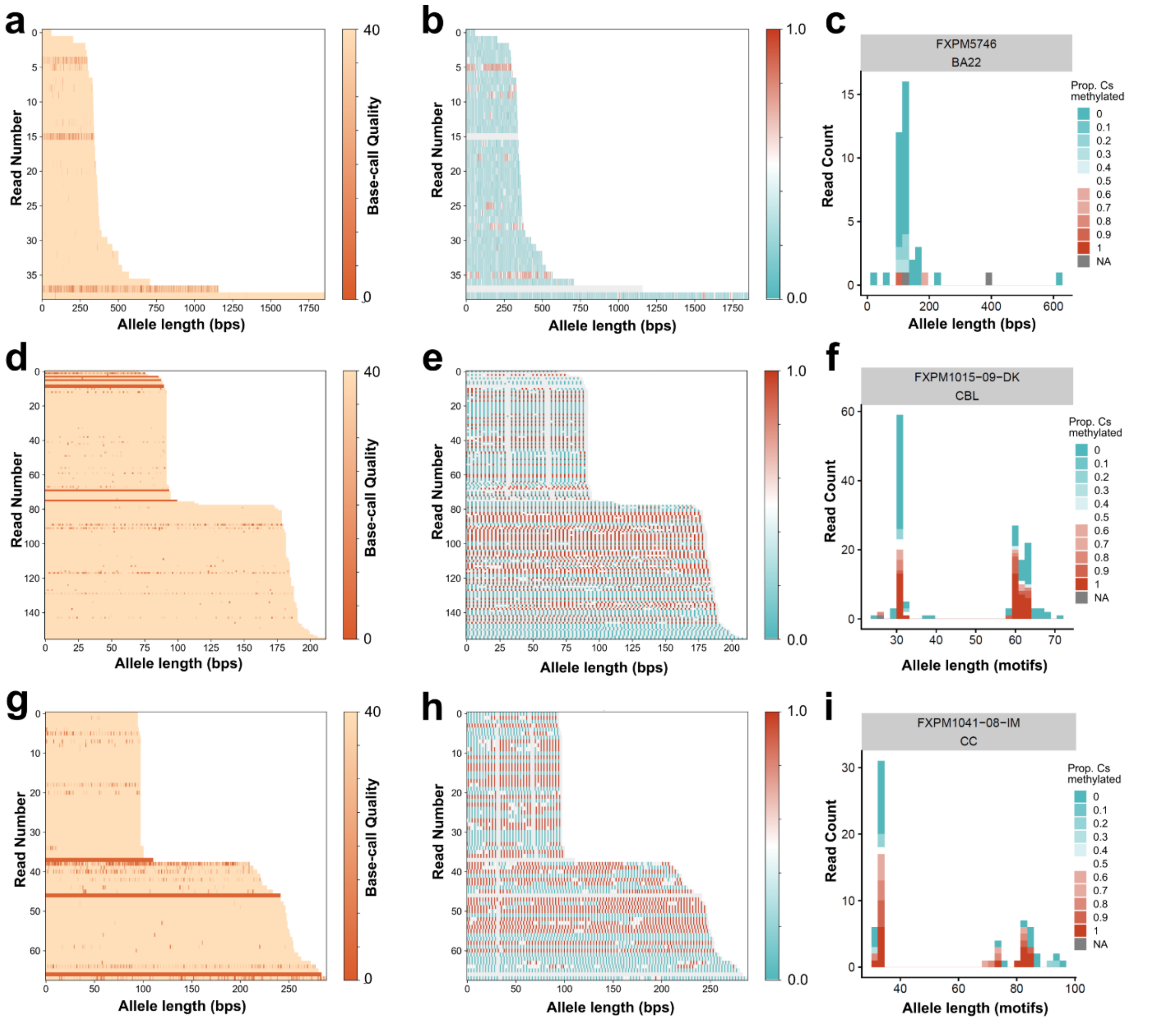

Supplemental Figure 5. Patterns of *FMR1* methylation in a larger FXTAS cohort. a-c) Base-call quality plot (a), waterfall methylation plot (b), and allele distribution plot (c) for BA22 of male FXPM 5746. **d-f)** Base-call quality plot (d), waterfall methylation plot (e), and allele distribution plot (f) for cerebellum of female FXPM 1015-09-DK. **g-i)** Base-call quality plot (g), waterfall methylation plot (h), and allele distribution plot (i) for corpus callosum (CC) of female FXPM 1041-08-IM.

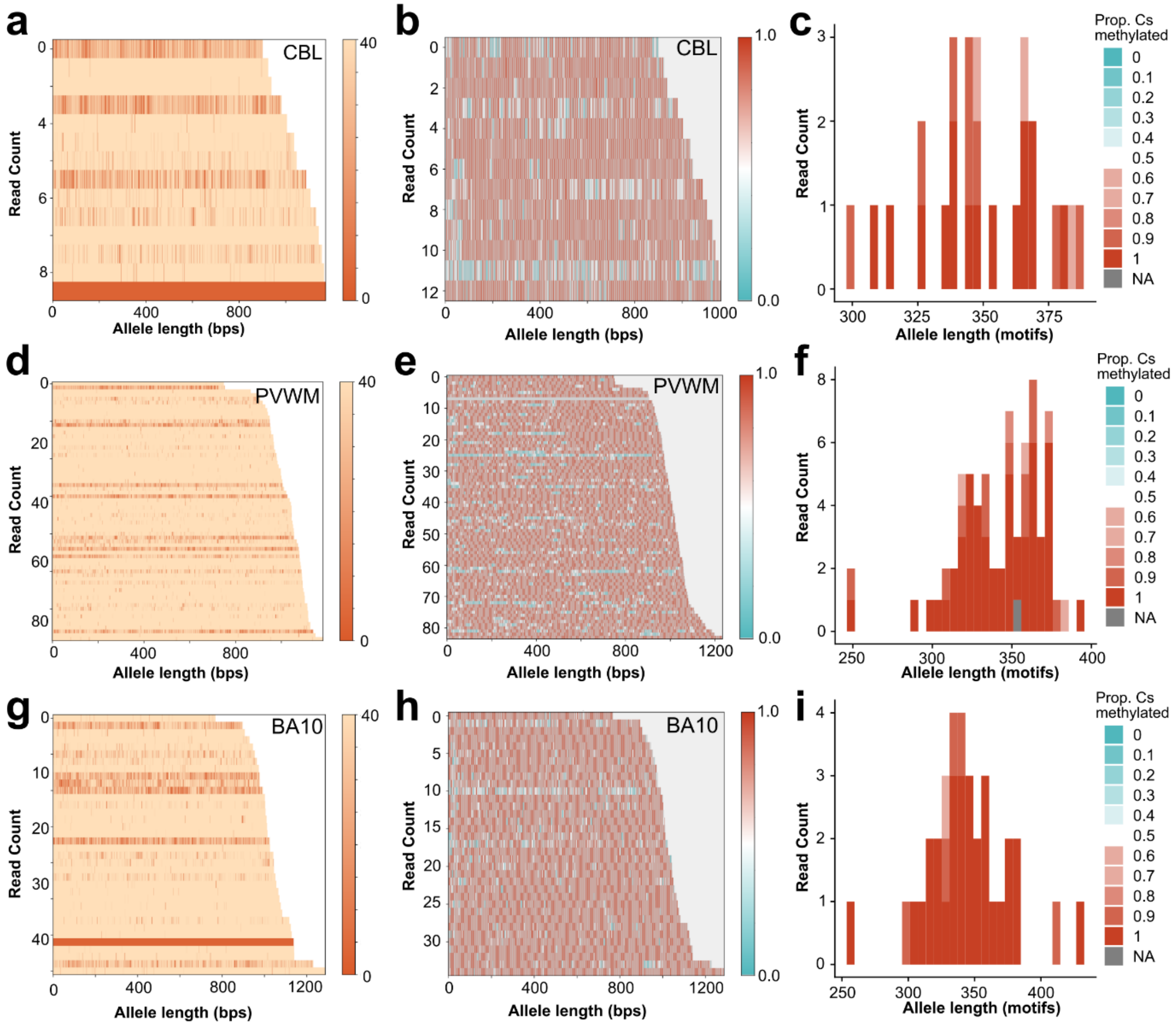

Supplemental Figure 6. Intra-individual patterns of *FMR1* methylation in FXS case. All plots are from case FXS 5319. **a-c)** Base-call quality plot (a), waterfall methylation plot (b), and allele distribution plot (c) for cerebellum (CBL). **d-f)** Base-call quality plot (d), waterfall methylation plot (e), and allele distribution plot (f) for periventricular white matter (PVWM). **g-i)** Base-call quality plot (g), waterfall methylation plot (h), and allele distribution plot (i) for BA10.

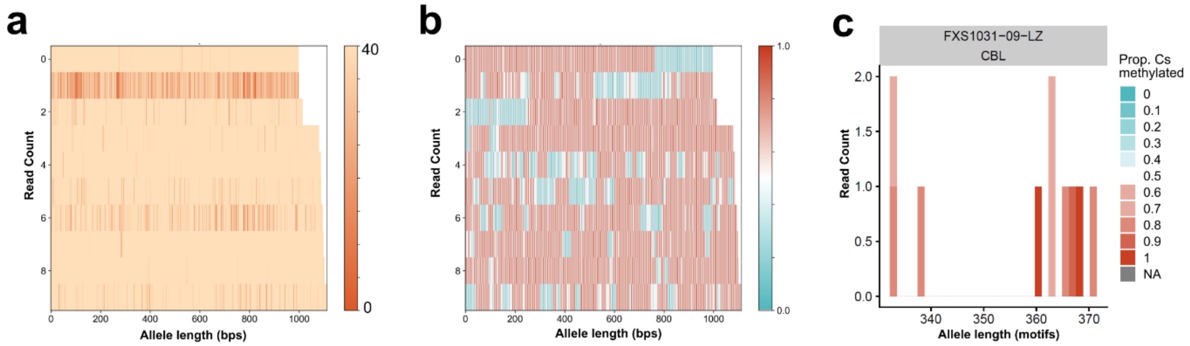

Supplemental Figure 7. Hypermethylation of *FMR1* is common in FXS. a-c) Base-call quality plot (a), waterfall methylation plot (b), and allele distribution plot (c) for cerebellum of male FXS 1031-09-LZ.
